# Novel 3,4-diarylpyrazole as prospective anti-cancerous agents

**DOI:** 10.1016/j.heliyon.2020.e04397

**Published:** 2020-07-14

**Authors:** Vivek Pandey, Garima Tripathi, Dhruv Kumar, Abhijeet Kumar, Pawan K. Dubey

**Affiliations:** a Centre for Genetic Disorder, Banaras Hindu University, Varanasi, U.P., India; b Department of Chemistry, T. N. B. College, TMBU, Bhagalpur, Bihar, India; c Amity Institute of Molecular Medicine & Stem Cell Research, Amity University Uttar Pradesh, Noida, India; d Department of Chemistry, School of Physical Sciences, Mahatma Gandhi Central University, Bihar, India

**Keywords:** Pyrazole, Molecular docking, Apoptosis, Reactive oxygen species, MDA-MB-231, Chemistry, Organic chemistry, Pharmaceutical chemistry, Biological sciences, Cell biology, Bioinformatics

## Abstract

Cancer is a leading cause of death globally. Despite therapeutic advancements the mortality rate of cancer is continuously increasing. Thus, it is important to identify and design potential therapeutic agents which can specifically bind with most common targets of cancer and inhibit tumor progression. The present work discloses the potential therapeutic application of the novel 3,4-diaryl 1*H*-pyrazoles as prospective anti-cancerous agent. The *in silico* molecular docking studies performed with 3,4-disubstituted pyrazoles as ligand with targets including DNA, BCL-2 and F1-ATP Synthase revealed strong binding affinity with DNA (-7.5 kcal/mol), BCL-2 (-8.1 kcal/mol) and F1-ATP Synthase (-7.2 kcal/mol). Furthermore, the *in silico* finding was validated with the *in vitro* cytotoxicity assay with human breast cancer cell line (MDA-MB-231). MDA-MB-231 cells treated with 3,4-diarylpyrazole resulted in an increase in annexin-V positive cells, production of reactive oxygen species (ROS), dissipation of the mitochondrial membrane potential and activation of caspase-3. Taken together, this study demonstrate that a novel synthesized 3,4-diarylpyrazoles, showed strong binding affinity against DNA, anti-proliferative activity and executed apoptosis through ROS-dependent caspase-3-mediated mitochondrial intrinsic apoptotic pathway against MDA-MB-231 cells. These findings increase our understanding of the molecular mechanism (s) by which 3,4-diarylpyrazoles can exert their anticancer activity and may contribute towards development of novel therapeutic agent against breast cancer.

## 1 Introduction

In general, the naturally occurring heterocyclic compounds along with their synthetic analogues are found to display a vast variety of biological activities [1, 2, 3, 4]. *N*-heterocycles such as functionalized pyroles, pyrimidines, pyridine, pyrazoles etc. are important bio-active heterocyclic scaffolds which are prevalent in nature and known to exhibit wide range of biological activities. The immense therapeutic importance of the *N*-heterocycles are evidenced by their presence as key pharmacophore in the major proportion of the commercially available and the best selling drugs prescribed to treat a vast range of diseases. Pyrazole derivatives have exhibited potential activity against a wide range of diseases such as anti-inflammatory, anti-bacterial, anti-cancerous, anti-viral and so on [2].

**Figure 1:**
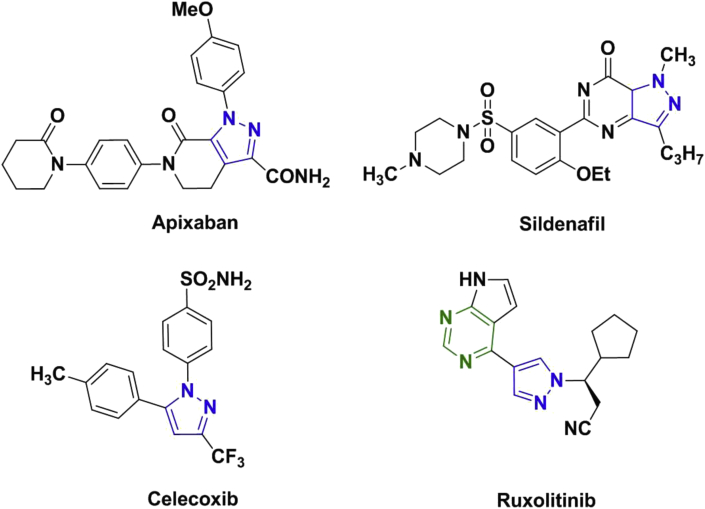
Examples of pyrazole scaffold in pre-existing drugs.

Some of the marketed drugs containing pyrazole scaffolds have been shown in Figure 1. For example, pyrazole containing drug, Apixaban is one of the most prescribed anti-coagulant [5]. Likewise, Sildenafil is a very popular drug with brand name Viagra used to treat erectile dysfunction in male [6]. Celecoxib and Ruxolitinib are anti-inflammatory and anti-cancerous drugs respectively containing pyrazole scaffolds [7, 8]. Apart from these examples, various other pyrazole based drugs are known in the literature and available in the market also [4]. In addition to that several research groups are involved in the synthesis of new pyrazole derivatives and exploration of their biological activities. For example, Liang and co-workers synthesized 4-Amino-(1*H*)-pyrazole derivatives and explored their efficacy as JAKs inhibitor comparable and some were found to be more potent than Ruxolitinib which is an approved anti-cancerous drugs [9]. Several other such novel pyrazoles derivatives are known to exhibit anti-cancerous activities [10]. Encouraged by the several such reports available in the literature about the effectiveness of pyrazole derivatives as potential anti-cancerous agents, the work reported herein explores the potential anti-cancerous activity of novel 3,4-diaryl pyrazoles.

## 2 Materials and methods

### 2.1 Synthesis of 3,4-diarylpyrazoles

**Figure 2:**
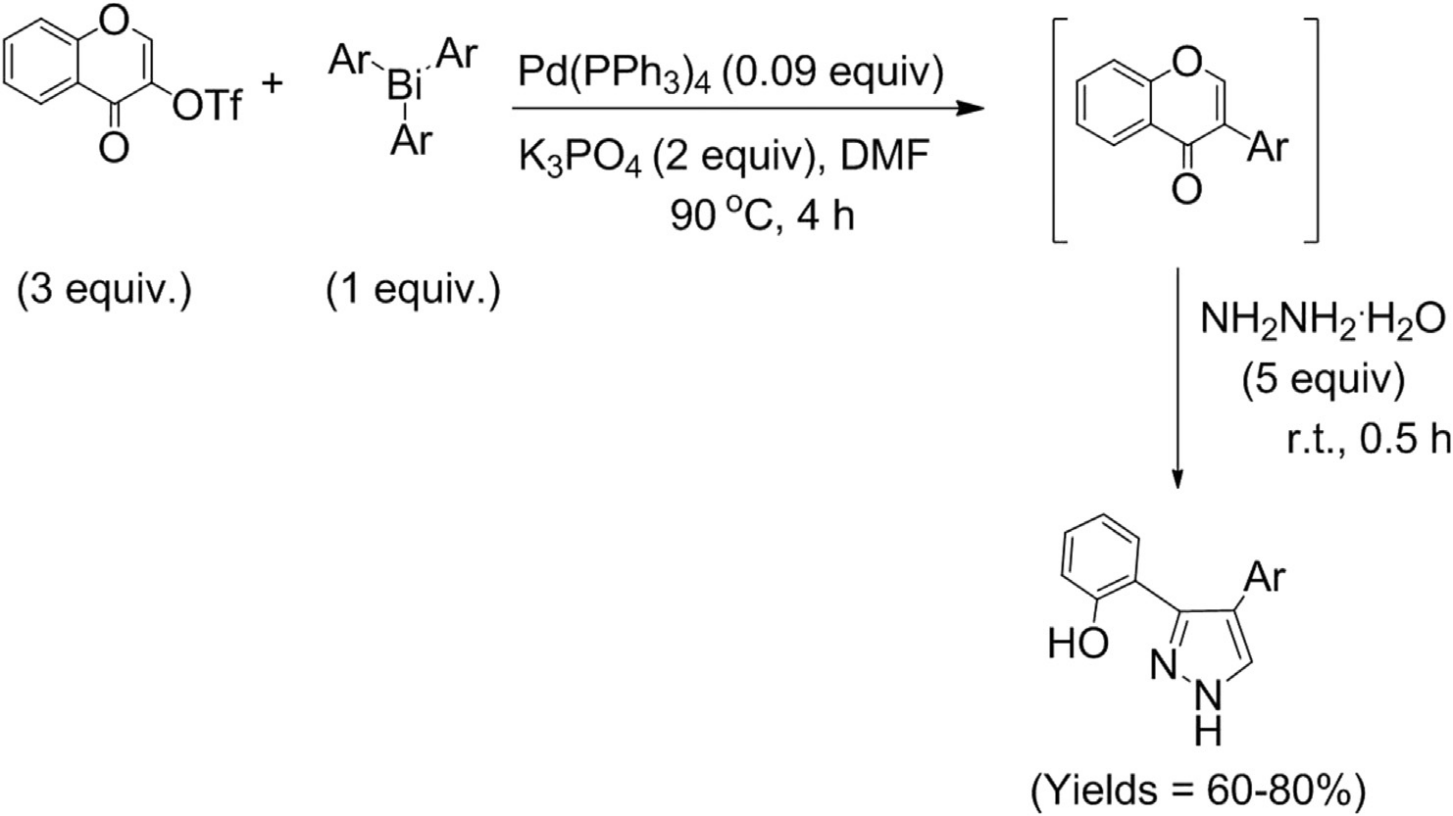
Synthesis of 3,4-diaryl pyrazoles under one-pot operation involving cross-coupling.

The 3,4-diarylpyrazoles was synthesized as described in detail earlier [4], following the reaction sequence presented in Figure 2 [4]. In detail, 3-trifloxychromone and triarylbismuths were used for the one-pot synthesis of 3,4-diarylpyrazoles as per described protocol [11, 12, 13]. Silica gel (100–200 mesh) was used for the isolation and purification of the final products. For thin layer chromatography, GF-254 silica gel (Merck) was used. In order to characterize the structure of the 3,4-diarylpyrazoles, JEOL-400 MHz (JNM ECS-400) spectrometer was used to record ^1^H and ^13^C NMR. Structures of the 3,4-diarylpyrazole were further confirmed through HRMS measurement using WATERS-Q-T of Premier-HAB213 and WATERS GCT Premier-CAB155 instruments. IR of the final products was measured using Bruker Vector 22 FTIR spectrometer. Standard drying procedures were followed to obtain dry solvents to be used for reaction and purification purposes.

### *2.2* *In silico* molecular docking studies

In order to investigate the potential anti-cancerous activity of these aryalted pyrazoles, molecular docking studies were performed. During the period of docking, the DNA & proteins were considered to be rigid and ligands were considered to be flexible. The configuration file for the grid parameters was generated using Auto Grid [14]. The docking study was also used to predict the type of amino acids in the active site of the proteins which interact with the diarylpyrazoles. The results less than 1.0 Å in positional root-mean-square deviation (RMSD) were considered ideal and clustered together for finding the favourable binding. In order to perform the molecular docking study, the ligands i.e. 3,4-diarylpyrazoles were prepared using Chem Sketch [15]. These were further converted into the PDB format from the file format of sdf with the help of Open Babel software [16]. In order to simplify the further analysis, ligands were converted to PDBQT format using the graphical user interface version of Autodock Tool 4 (ADT) [14]. Similarly, the atomic coordinates of the DNA (PDB ID-1D66), BCL-2 (PDB ID-1G5M) and F1-ATP Synthase (PDB ID-1BSN) were downloaded from the RCSB PDB database. Before docking, charges, solvation parameters and fragmental volumes to the DNA, BCL-2 and F1-ATP Synthase were assigned using the Autodock Tool 4 (ADT). Further, to simplify the analysis, the DNA and proteins were also saved to PDBQT format.

### 2.3 Biological studies

Encouraged by the results from molecular docking study which revealed considerable binding affinity of pyrazole derivative with the targets; this compound was further taken forward to investigate its efficacy as potential anti-cancerous agent. Chemicals used for these studies were procured from Sigma Chemical Co. (St. Louis, MO) unless stated otherwise. The cell line (MDA-MB-231; human breast cancer) was procured from National Centre for Cell Science (NCCS), Pune, India.

### 2.4 Preparation of culture medium and working concentration of synthesized 3,4-diarylpyrazoles

The cells were maintained in high glucose containing Dulbecco's Modified Eagle Medium (DMEM) fortified with 10% fetal bovine serum (FBS) and antibiotics (100 units/mL penicillin and 100 μg/mL streptomycin). To get the working concentrations (2.5, 5, 10, 20, 40, 80, 120, 180 and 250 μM), stock solution (10 mM in DMSO) of synthesized 3,4-diarylpyrazoles were diluted in a freshly prepared working DMEM. The osmolarity (290 ± 5 mOsmol) and pH 7.2 ± 0.10 of the culture medium did not change after adding 3,4-diarylpyrazoles. The culture medium without 3,4-diarylpyrazoles was used as control.

### 2.5 MTT assay

To examine cytotoxicity of synthesized 3,4-diarylpyrazoles, the colorimetric MTT metabolic assay were performed. In this study, MDA-MB-231 cell line was seeded (10,000 cells/well) in 24-well tissue culture plates for 24 h. After that cells were incubated with different concentrations of synthesized 3,4-diarylpyrazoles (2.5, 5, 10, 20, 40, 80, 120, 180 and 250 μM) for 24 h in a CO_2_ incubator [17]. The working concentration of MTT (5 mg/mL) was prepared in PBS, and 10μL was added into each well; after 4 h incubation, 100μL of DMSO was used to dissolve formazan crystals. The absorbance intensity was taken using a microplate reader (BioRAD 680, USA) at 590 nm. The effective concentration (EC_50_ value) were calculated with help of GraphPad prism 4.0 (GraphPad software, USA) using non-linear regression analysis. The experiments were repeated at least three times. The cell survival percentage was calculated using the following formula:

%Control=[Mean O. D. of the Drug treated well/Mean O. D. of the control well]×100

### 2.6 Acridine orange/propadium iodide (AO/PI) dual staining assay

The AO/PI staining was used to examine morphological changes in MDA-MB-231 cells after 3,4-diarylpyrazoles treatment. In brief, MDA-MB-231cells (1×10^6^) were treated with or without an optimum effective dose of 3,4-diarylpyrazoles (50 μM) for 24 h in the CO_2_ incubator. After washing in PBS, cells were further incubated with 100μL of AO/PI solution (1 part of 100 μg/mL of AO in PBS; 1 part of 100 μg/mL of PI in PBS). After 30 min incubation, cells were washed with PBS and morphology examined under a fluorescence microscope (EVOS FL, Life technologies). Viable cells exhibit intact membrane and produces green fluorescence while apoptotic cells show bright orange color which indicates dead cells. The photographs were captured using inverted fluorescence microscope (EVOS FL, Life technologies at 20x magnification).

### 2.7 Apoptosis detection assay by annexin-V/PI

To validate our AO/PI data, 3,4-diarylpyrazoles (50 μM) treated MDA-MB-231 cells were stained with Annexin V/PI and FACS analysis were carried out. In detail, after treatment, the MDA-MB-231 cells were incubated with 2μl of annexin V-FITC and 5μl of PI (100 μg/ml) in 200μl of binding buffer at room temperature for 15 min in the dark. After washing in 1X PBS, stained cells were examined by flow cytometry using BectoDickinson fluorescence-activated cell sorter (FACS) IV Calibur (Becton Dickinson, San Jose, CA, USA). The flow cytometry data were analyzed and plotted for annexin V-FITC using a Cell Quest software (Becton Dickinson).

### 2.8 Analysis of ROS level

To examine whether apoptosis is mediated by ROS production, after 24 h of 3,4-diarylpyrazoles (50 μM) treatment, the MDA-MB-231 cells were washed and incubated with 10μM dichlorofluorescein diacetate (DCFH-DA, Beyotime, Nantong, China) at 37 °C for 30 min. Finally cells were washed 3 times with PBS and examine under fluorescence microscope (EVOS FL, Life technologies) as per manufacturer's instructions. The fluorescence of DCF was detected at an excitation wavelength of 488 nm and an emission wavelength of 525 nm using inverted fluorescence microscope (EVOS FL, Life technologies).

### 2.9 Assessment of mitochondrial membrane potential

The mitochondrial membrane potential was analyzed using flurochrome reporter JC-1 (1μM) dye (5,5,6,6-tetra-chloro-1,1,3,3-tetra-ethyl-benz- imidazolo-carbocyanine iodide) (BD Bioscience) as per manufacture's instruction. In brief, cells were treated with 3,4-diarylpyrazoles (50 μM) for 24 h and then cells were incubated with JC-1 dye (1μM) for 10 min. Photographs were taken using an inverted fluorescence microscope (EVOS FL, Life technologies) and imaged first under red filter and then under green filter. Red fluorescence indicated cells with intact mitochondria while green fluorescence indicated cells with depolarized mitochondria. Values are expressed as total red CTCF from three replicates.

### 2.10 Immunocytochemistry of Bcl_2_ and caspase 3

The immunocytochemistry was performed to detect the protein expression of anti-apoptotic protein Bcl_2_ and apoptotic protein caspase3 in treated MDA-MB-231 cells. Briefly, 3,4-diarylpyrazoles (50 μM) treated MDA-MB-231 cells were fixed using 4% para-formaldehyde for 10 min, after PBS wash, cells were incubated with sodium citrate buffer for 30 min. To avoid background staining, the cells incubated with 5% blocking serum for 20 min. After PBS wash, cells were incubated with primary monoclonal antibodies Bcl_2_ and caspase3 (1:200; Santacruze Biotechnology, Inc) overnight at 4 °C. After incubation, cells were washed with PBS, and then incubate with texas red anti-mouse (1:800) secondary antibodies for 2h at 37 °C. The photographs were captured using inverted fluorescence microscope (EVOS FL, LifeTechnologies).

### 2.11 Real-time PCR (quantitative qPCR)

The quantitative real-time PCR was performed to examine mRNA expression level of apoptotic genes (Caspase-3) and anti-apoptotic gene (Bcl_2_) in 3,4-diarylpyrazoles (50 μM) treated MDA-MB-231 cells. The primer sequence of genes which used in this study was shown in Table 1. In brief, after 24 h s of treatment, MDA-MB-231 cells were harvested, total RNA was isolated through trizol reagent and reverse transcribed according to manufacturer's protocol (Thermo scientific). qPCR was then performed with SYBR green dye using Applied Biosystems™Real-Time PCR Instruments (Life Technologies, Carlsbad, CA) according to manufacturer's instruction. The changes in mRNA expression level were calculated using the Relative Quantity (RQ) equation, RQ = 2-^ΔΔ^CT; where delta cycle threshold (ΔCT) denoted a difference in CT values between the target gene and GAPDH, and ΔΔCT signified the differences in the ΔCT value of treated sets compared to control.

**Table 1:** List of used primers sequence.

Gene	Forward Primer	Reverse Primer
GAPDH	GGGCATCCTGGGCTACACTGA	TCCACCACCCTGTTGCTGTAG
BCL 2	GTGGATGACTGAGTACCT	CCAGGAGAAATCAAACAGAG
Caspase-3	GTAGATGGTTTGAGCCTGAG	CCAGTGCGTATGGAGAAATG

### 2.12 Statistical analysis

Data obtained in this study were analyzed using SPSS statistical software (SPSS, Inc., Chicago, IL) and expressed as means ± SEM from three experiments. The ImageJ software (version 1.44 from the National Institute of Health, Bethesda, USA) was used to analyze intensity of fluorescence. A probability of p < 0.05 was considered as statistically significant.

## 3 Results and discussion

### 3.1 Preparation of 3,4-diarylpyrazoles

The 3,4-diarylpyrazoles used to study their prospective anti-cancerous properties were earlier synthesized by Kumar et. al [4]. following the reaction protocol shown in Figure 2. The synthesis involves the in situ formation of isoflavones under Pd-catalyzed reaction cross-coupling method performed with 3-trifloxychromone and triarylbismuth followed by reaction with hydrazine hydrate to furnish phenol substituted C-arylated pyrazoles in one pot operation in good to excellent yield.

### 3.2 Molecular docking studies

**Figure 3:**
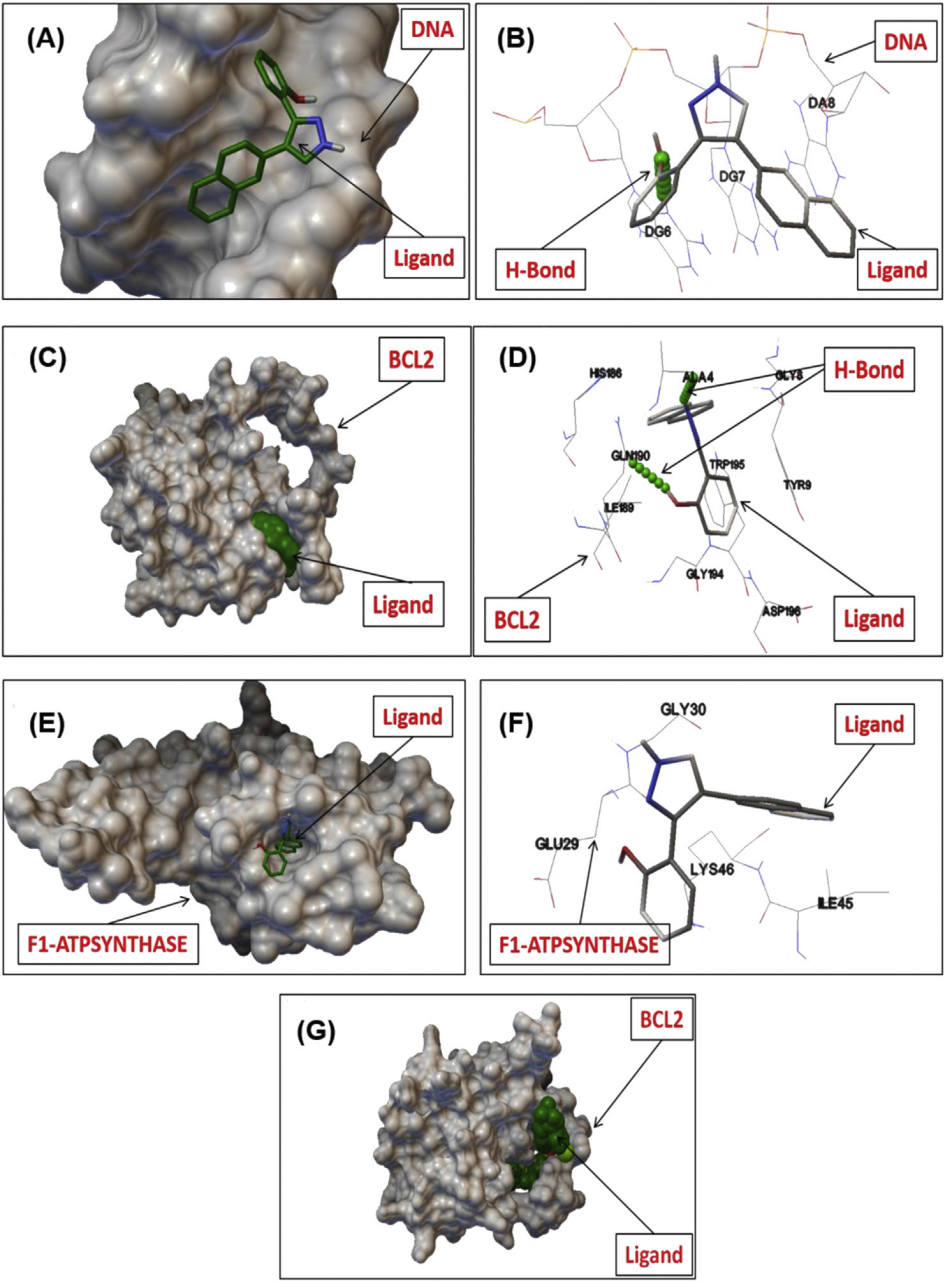
The 3D views of interaction of pyrazole (1.6) with DNA (A & B),BCL-2 (C & D), F1-ATP Synthase (E & F) and Navitoclax (Known BCL-2 inhibitor) with BCL-2 (G).

Among all the ligands, 2-(4-(naphthalen-2-yl)-1*H*-pyrazol-3-yl) phenol (1.6) showed strong binding affinity with DNA (-7.5 kcal/mol), BCL-2 (-8.1 kcal/mol) and F1-ATP Synthase (-7.2 kcal/mol) (Table 2). Whereas the known BCL-2 inhibitor, Navitoclax showed -8.9 kcal/mol binding affinity with BCL-2 (Figure 3), which is close to the diarylpyrazoles. The top ten docking result generated using Autodock-Vina, which showed best binding conformations of the ligands with receptors at their active site. In past few years, the molecular docking studies have extensively been used as an imperative tool in the area of drug discovery as it allows to investigate the probable non-covalent interactions between the ligands which are generally small molecules and macromolecules such as proteins, enzymes, DNA etc. as targets [18]. Using the command line version of Autodock Vina [19] molecular docking study was also performed in present case to realize the effect of functionalized pyrazoles on reactive oxygen species (ROS) production through their binding interaction with DNA, BCL-2 and F1-ATP Synthase (Table 2). The 3D view of DNA, BCL-2 and F1-ATP Synthase with ligand interactions of the best confirmation created by ADT are shown in molecular surface representation (Figure 3). As clearly shown in Figure 3, the establishment of important interactions between ligand and the amino acid residues which are involved in the catalytic mechanism of ROS generation through DNA, BCL-2 and F1-ATP Synthase. The protein-ligand complex is stabilized mainly by electrostatic interactions, hydrogen bonding and ionic interactions. The top docked confirmation generated by each docking showed well established interaction with one or more amino acids at the catalytic domains of DNA and proteins. The top-ranked pose with lowest binding affinities is generally used as a standard selection in most of the docking programs.

**Table 2:** Results of molecular docking studies between 3,4-diarylpyrazoles and onco-proteins.

S.No.		DNA	ERBB2/HER2	BCL2∗	F1-ATPSYNTHASE
1	Ar = Ph **1.1**	-6.6	-8.2	-6.7	-6.4
2	Ar = *p*-tolyl **1.2**	-6.8	-8.5	-7.1	-6.6
3	Ar = 4-methoxyphenyl **1.3**	-6.6	-7.8	-6.7	-6.1
4	Ar = 4-chlorophenyl **1.4**	-6.2	-8.4	-6.8	-6.2
5	Ar = 3-methoxyphenyl **1.5**	-6.4	-7.5	-6.9	-6.4
6	Ar = 2-naphthyl **1.6**	**-7.5**	**-9.8**	**-8.1**	**-7.2**
7	Ar = 4-fluorophenyl **1.7**	-6.7	-8.3	-6.9	-6.5

### 3.3 Biological studies

We investigated whether the synthesized 3,4-diarylpyrazoles exert anticancer activity against breast cancer, and if so, via what mechanism. Breast cancer is one of the leading cancers in women world-wide. From last once decade, several medical interventions like surgery, chemotherapy and radiotherapy has been used to treat breast cancer [20, 21]. Over the past several years, many of the chemical compounds having *N*-heterocycles such as pyridine, triazole, pyrazole, pyrimidines scaffolds along with *O*-heterocycles such as benzofuran, coumarin, chromones etc have increased significant notice because of their safety, efficacy, low-cost, and therapeutical potential against cancers.

**Figure 4:**
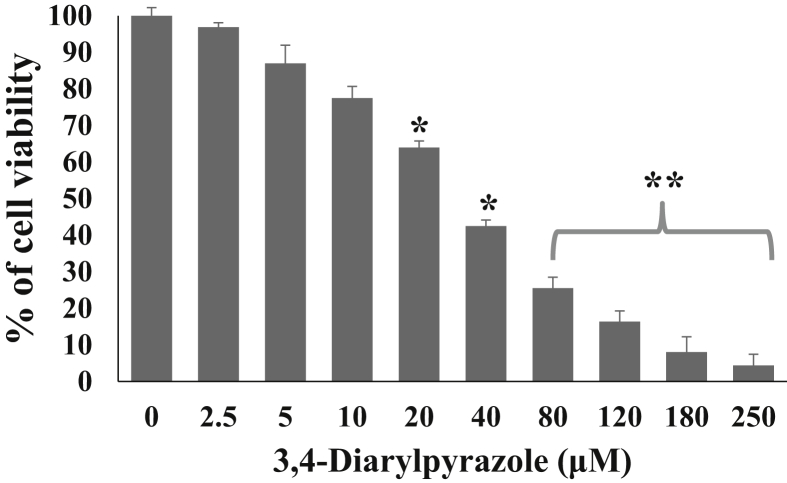
Cell viability of MDA-MB-231cells treated with 3,4-diarylpyrazoles. The bar diagram showing cell viability of MDA-MB-231cells after treatment with 0.0, 2.5, 5, 10, 20, 40, 80, 120, 180 and 250 μM of 3,4-diarylpyrazoles for 24 h. Data are normalized with control group (medium only) and presented as mean ± SEM.

**Figure 5:**
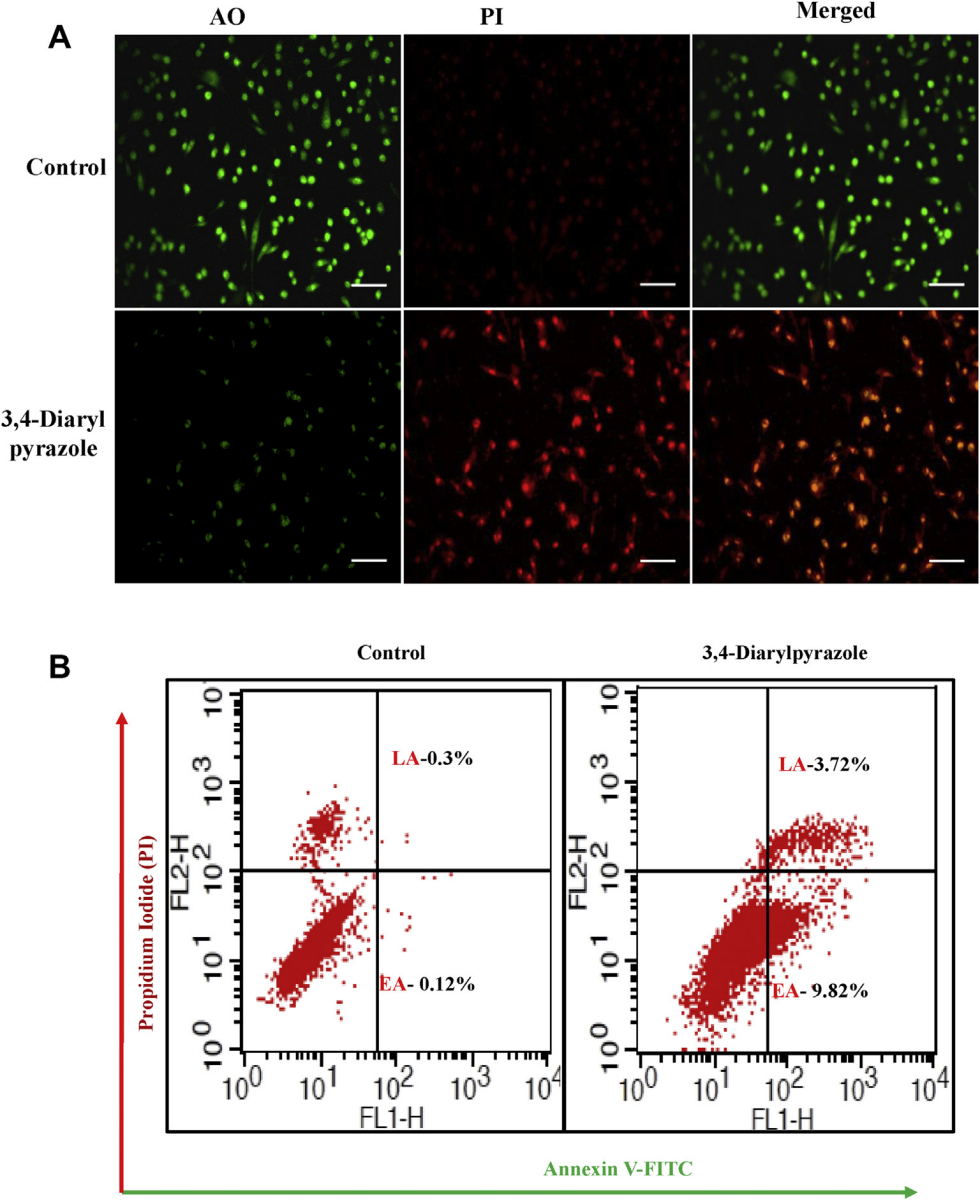
AO/PI staining and flowcytometric analysis of MDA-MB-231cells treated with/without 3,4-diarylpyrazoles **(A)** The untreated cells are showing green fluorescence (AO)/representing live cells while treated cells showing red fluorescence (PI) representing dead cells **(B)** flow cytometry data are showing that treatment of 3,4-diarylpyrazoles (50 μM) induces early apoptotic as well as late apoptotic in MDA-MB-231 cells. (Bar = 200 μm).

**Figure 6:**
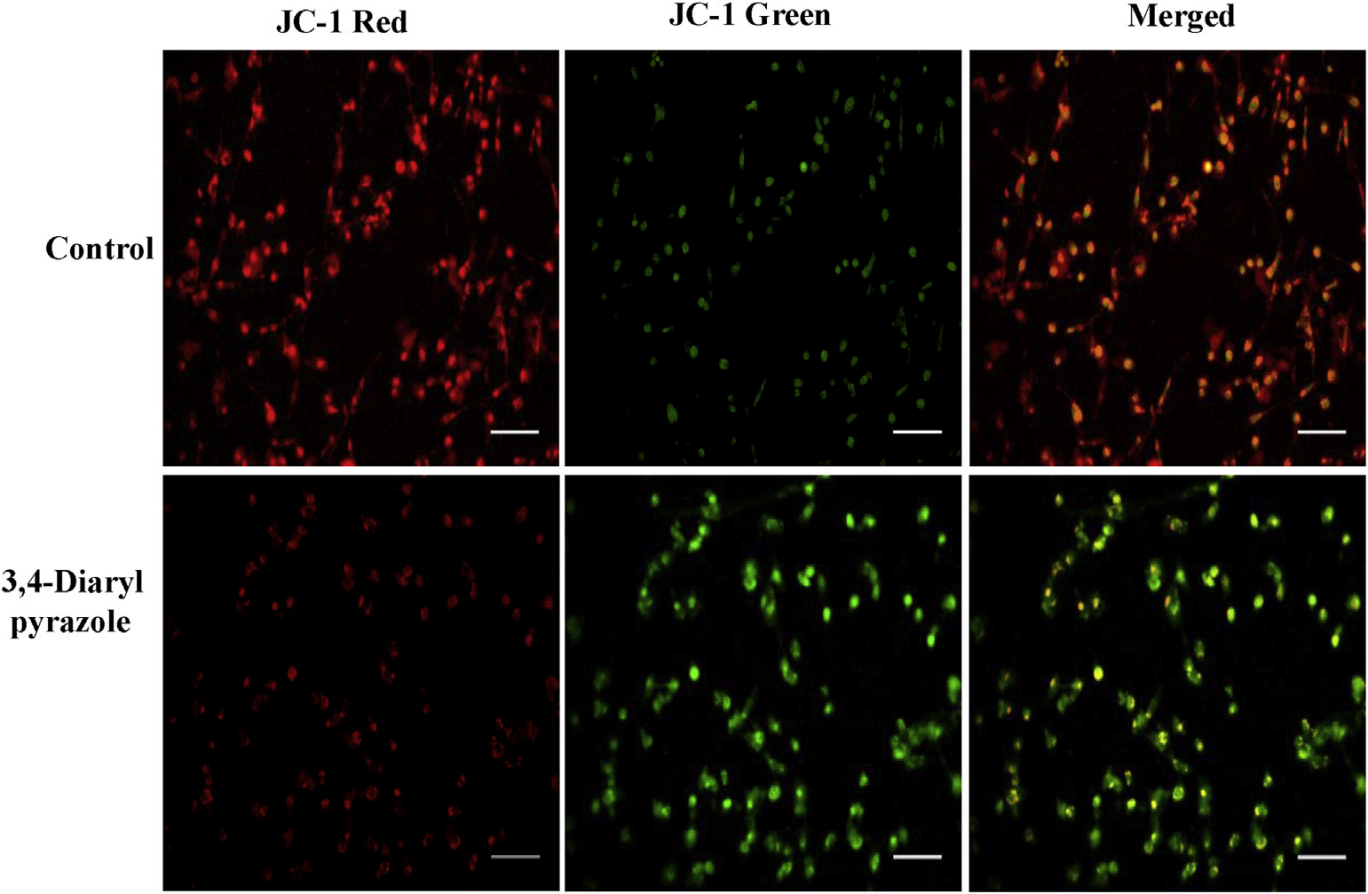
Representative photographs showing mitochondrial membrane potential in 3,4 diarylpyrazoles treatment in MDA-MB-231cells. Bar diagram shows CTCF analysis of fluorescence intensity of control and treated cells. Three independent experiments were conducted to confirm the results. (Bar = 100 μm).

As shown in Figure 4, the metabolic activity of MDA-MB-231 cells were inhibited by 3,4-diarylpyrazoles in a dose dependent manner. We found half maximal inhibitory concentration (IC_50_) of 3,4-diarylpyrazoles was 50 μM after 24h. Further, to examine whether the cytotoxic effect of 3,4-diarylpyrazoles in MDA-MB-231 cells was associated with apoptosis induction, various assays has been conducted. As shown in Figure 5A and 5B, the 3,4-diarylpyrazoles induces morphological features of apoptosis as it increased population of PI positive cells (early apoptosis) in AO/PI dual-staining assay and also increased the number of Annexin-V/PI positive cells (late apoptotic cells) in FACS assay as compared to respective control. Our results suggest that the synthesized compound possess tremendous capacity to inhibit proliferation and induction of apoptosis in MDA-MB-231 cells. Further, to assess the status of mitochondrial membrane potential (ΔΨm) in response to 3,4-diarylpyrazoles treatment, the cells were stained with JC-1 fluorochrome. High fluorescence intensity (higher JC-1 green fluroscence) was observed in treated cells which is indicative of depolarized ΔΨm state of mitochondria (Figure 6). This result suggests that 3,4-diarylpyrazoles induced mitochondrial membrane depolarization may associated with increase of oxidative stress or/reactive oxygen species (ROS).

**Figure 7:**
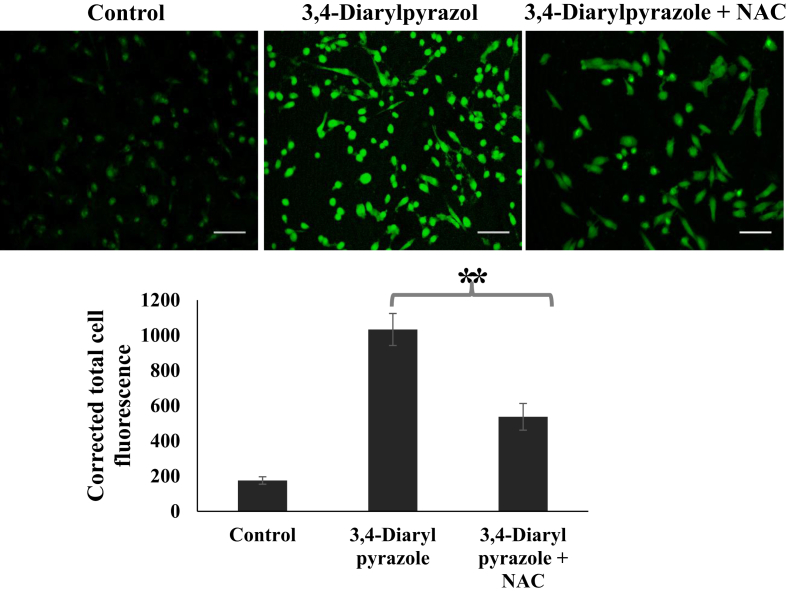
Representative photographs showing intracellular ROS level in 3,4-diarylpyrazoles treatment in MDA-MB-231cells. Significant increased ROS level was noticed in treated MDA-MB-231 cells. The bar diagram shows CTCF analysis of fluorescence intensity of control and treated cells. Three independent experiments were conducted to confirm the results. (Bar = 200 μm).

It has already been demonstrated that many cytotoxic agents/compounds inhibits growth of tumor through ROS dependent activation of apoptosis [22]. It has also been shown that increased level of ROS in cancer cells in response to phytochemicals eventually leads to cell death [23]. In present study, to examine whether 3,4-diarylpyrazoles induced apoptosis is mediated by production of ROS. Intracellular ROS level was analyzed in 3,4-diarylpyrazoles treated and untreated cells using a fluorescent probe DCFH-DA. As shown in Figure 7, significantly increased DCFDA fluorescence intensity was observed at 50 μM of 3,4-diarylpyrazoles as compared to control (P < 0.05). Furthermore, the co-treatment of N-acetyl cysteine (NAC) which protects cells against ROS-induced damage, almost suppressed the 3,4-diarylpyrazoles induced ROS generation in MDA-MB-231 cells (Figure 7). Lower fluorescence intensity was observed in combination with NAC +3,4-diarylpyrazoles as compared to 3,4-diarylpyrazoles alone suggest that 3,4-diarylpyrazoles induced ROS generation, which finally induced apoptosis in MDA-MB-231 cells. However, mechanism by which anti-cancer drugs may cause elevated ROS levels varies in response to treatment, such as disruption of the mitochondrial electron transport chain, changes in mitochondrial membrane potential and dysregulation of antioxidant enzymes.

It is reported that apoptosis is mediated by mitochondrial ROS production which may activate both the intrinsic and extrinsic pathways of apoptosis [24, 25]. In this study, our synthesized compound 3,4-diarylpyrazoles significantly induce ROS generation in MDA-MB-231 cells, indicate that 3,4-diarylpyrazoles induced ROS-dependent intrinsic pathway of apoptosis. Although, ROS-independent mechanism of cell death was also reported in some other types of cells [26, 27] which suggests that some other intrinsic pathways are also involved to cell death because ROS is not always necessary to induce apoptosis.

**Figure 8:**
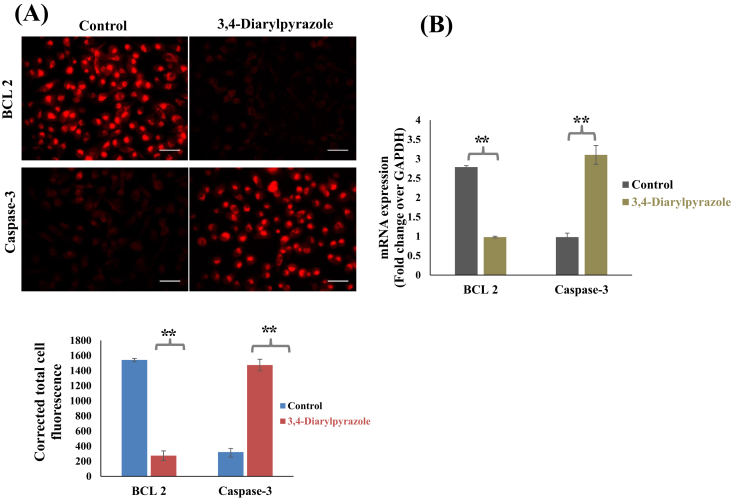
Effect of 3,4-diarylpyrazole in apoptosis induction on MDA-MB-231 cells **(A)** Representative photographs showing expression level of anti-apoptotic (Bcl_2_) and pro-apoptotic (Caspase-3) protein in 3,4-diarylpyrazoles treated MDA-MB-231 cells. **(B)** The relative fold change expression of Bcl_2_ and caspase-3 protein in 3,4-diarylpyrazoles treated MDA-MB-231 cells. The graph represents means ± SEMs from at least 3 separate experiments. The asterisk (∗∗) indicates a significant P value the significance level in comparison to untreated group; ∗∗P < 0.05.

**Figure 9:**
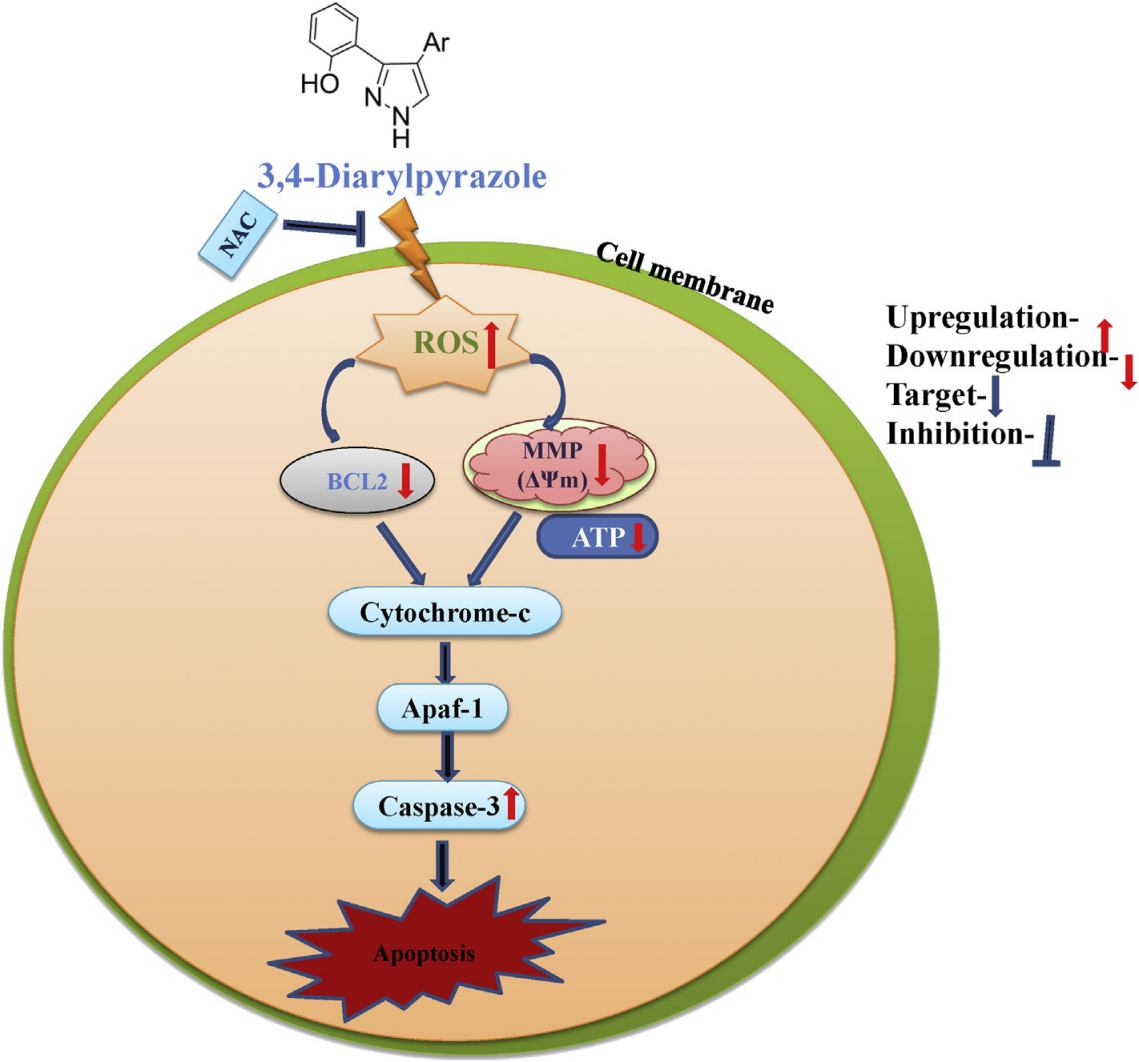
The proposed model diagram represented the molecular targets of 3,4-diarylpyrazoles on MDA-MB-231 cells. The 3,4-diarylpyrazoles induced ROS by disrupting mitochondrial membrane potential which release caspase-3 and thereby apoptosis.

To delineate the molecular mechanism of apoptosis, the expression level of BCL-2 and caspase-3 in 3,4-diarylpyrazoles treated MDA-MB-231 cells were examined by immunocytochemistry and real-time PCR. In general, the ratio of the pro-apoptotic gene (Bax) and anti-apoptotic gene (BCL-2) are detrimental factors for programmed cell death, including apoptosis. It is reported that caspase-3 is activated death proteases that finally leads to DNA fragmentation [28]. As shown in Figure 8A, total corrected cell fluorescence intensity level of apoptotic Caspase-3 protein were found higher in 3,4-diarylpyrazoles treated UL cells as compared to level of BCL-2 protein. It suggest that 3,4-diarylpyrazoles significantly suppressed the expression Bcl_2_ whereas it induced caspase-3 expression in treated MDA-MB-231 cells as compared to control (Figure 8A). Further, quantitative RT-PCR analysis showed higher (p < 0.05) mRNA expression of pro-apoptotic gene caspase-3 whereas down regulation of Bcl_2_ mRNA expression in 3,4-diarylpyrazoles treated MDA-MB-231 cells as compared to control (Figure 8B). These results further suggest that 3,4-diarylpyrazoles induces apoptosis in MDA-MB-231 cells via ROS-dependent caspase-3-mediated mitochondrial intrinsic apoptotic pathway (Figure 9).

## 4 Conclusion

This study first time demonstrated that a novel synthesized 3,4-diarylpyrazoles, exerted strong binding affinity against DNA, potent anti-proliferative activity and executed apoptosis through ROS-dependent caspase-3-mediated mitochondrial intrinsic apoptotic pathway against MDA-MB-231 cells. Our results may help to increase understanding of the molecular mechanisms by which 3,4-diarylpyrazoles inducing cell death in MDA-MB-231 cells and may contribute towards development of novel therapeutic agent against breast cancer.

## Declarations

### Author contribution statement

P. Dubey and A. Kumar: Conceived and designed the experiments; Analyzed and interpreted the data; Wrote the paper.

V. Pandey and D. Kumar: Performed the experiments; Analyzed and interpreted the data; Contributed reagents, materials, analysis tools or data.

G. Tripathi: Performed the experiments; Analyzed and interpreted the data; Contributed reagents, materials, analysis tools or data; Wrote the paper.

### Funding statement

This study was financially supported by 10.13039/501100001843Science and Engineering Research Board, Ministry of Science and Technology, Government of India (Grant No. SERB/YS/000819).

### Competing interest statement

The authors declare no conflict of interest.

### Additional information

Suport of Prof. M.L.N. Rao and research lab facilities at IIT-Kanpur is dully acknowledged.
